# Early Acute Kidney Injury in Allogeneic Hematopoietic Cell Transplantation: Incidence, Severity, and Associated Factors in a 634‐Recipient Cohort

**DOI:** 10.1111/ejh.70227

**Published:** 2026-05-21

**Authors:** Luiz Carlos da Costa‐Junior, Rita de Cássia Barbosa da Silva Tavares, Marina Izu, Simone Cunha Maradei, Maria Claudia Rodrigues Moreira, Marta Colares Nogueira, Décio Lerner, Fabrício Guimarães Bino, Patrícia Moriel, Teresa de Souza Fernandez, Paulo Caleb Júnior Lima Santos

**Affiliations:** ^1^ Postgraduate Program in Medical Sciences Faculdade de Medicina da Universidade de São Paulo (FMUSP) São Paulo Brazil; ^2^ Bone Marrow Transplant Center Instituto Nacional de Câncer (INCA) Rio de Janeiro Brazil; ^3^ Bone Marrow Transplant Program Hospital Universitário Clementino Fraga Filho (HUCFF), Universidade Federal do Rio de Janeiro (UFRJ) Rio de Janeiro Brazil; ^4^ Escola de Ciências da Saúde Afya Universidade Unigranrio Duque de Caxias Brazil; ^5^ Division of Nephrology Hospital Universitário Clementino Fraga Filho (HUCFF), Universidade Federal do Rio de Janeiro (UFRJ) Rio de Janeiro Brazil; ^6^ Faculdade de Ciências Médicas Universidade Estadual de Campinas (Unicamp) Campinas Brazil; ^7^ Cytogenetic Laboratory, Cell and Gene Therapy Program Instituto Nacional de Câncer (INCA) Rio de Janeiro Brazil; ^8^ Department of Pharmacology Escola Paulista de Medicina, Universidade Federal de São Paulo (EPM‐Unifesp) São Paulo Brazil

**Keywords:** acute kidney injury, calcineurin inhibitors, early complications, hematopoietic cell transplantation, pharmacokinetics

## Abstract

**Background:**

Acute kidney injury (AKI) is a frequent and clinically relevant complication in allogeneic hematopoietic cell transplantation (HCT) recipients, particularly during the early post‐HCT period. Although AKI has been extensively reported in long‐term follow‐up studies, data focusing on the initial hospitalization phase remain limited, despite its potential impact on subsequent clinical outcomes.

**Objective:**

To evaluate the incidence, severity, and clinical and transplant‐related determinants of AKI during the first 3 weeks after allogeneic HCT.

**Methods:**

We conducted a retrospective cohort study including 634 consecutive recipients undergoing allogeneic HCT between 2002 and 2023. AKI was defined according to KDIGO criteria and assessed from day +1 to day +21. Cumulative incidence was estimated, and associations were evaluated using Cox proportional hazards models. Pre‐AKI exposure to calcineurin inhibitors was analyzed.

**Results:**

By day 21, AKI occurred in 346 patients (55%), and 27% of cases reached KDIGO stages 2–3. Median time to AKI was 12 days, with earlier onset among stage 3 events (median 5 days). In multivariable analysis, AST > 40 U/L was independently associated with an increased risk of any‐stage AKI (HR 1.58, 95% CI 1.17–2.12; *p* = 0.003), whereas albumin > 3.5 g/dL was protective (HR 0.56, 95% CI 0.44–0.71; *p* < 0.001). Severe AKI was associated with AST > 40 U/L (HR 1.93, 95% CI 1.08–3.44; *p* = 0.025) and year of transplantation, with higher risk observed in earlier categories (2002–2008: HR 3.68, 95% CI 1.75–7.72; *p* < 0.001), while higher albumin levels remained inversely associated (HR 0.42, 95% CI 0.26–0.70; *p* < 0.001). Higher pre‐AKI cyclosporine trough levels (HR 1.11, 95% CI 1.05–1.17; *p* < 0.001) and a greater proportion of supratherapeutic days (HR 1.06, 95% CI 1.02–1.10; *p* = 0.005) were also associated with AKI.

**Conclusion:**

Early AKI in HCT recipients is strongly associated with baseline clinical vulnerability, hepatic dysfunction, and calcineurin inhibitor exposure. Elevated AST and lower albumin levels identify patients at increased risk, while higher cyclosporine exposure contributes to early renal injury. In contrast, transplant‐related characteristics show limited independent influence. These findings support early risk stratification, careful therapeutic monitoring, and individualized immunosuppression to reduce renal complications during the initial post‐HCT period.

## 1 Introduction

Hematopoietic cell transplantation (HCT) is a potentially curative therapeutic modality for a wide range of malignant and non‐malignant hematologic diseases [1, 2]. Despite advances in peri‐transplant care and the increasing number of procedures performed over recent decades [3, 4], HCT recipients remain susceptible to infections and non‐infections complications [5, 6], particularly during the early post‐HCT period, which is characterized by prolonged hospitalization and high clinical complexity [7, 8, 9].

Acute kidney injury (AKI) is a non‐infectious complication associated with HCT and may occur in both early and late post‐transplant phases [10, 11]. According to the *Kidney Disease: Improving Global Outcomes* (KDIGO) criteria [12], AKI is defined based on changes in serum creatinine and/or urine output. From a pathophysiological perspective, AKI may involve prerenal, intrinsic renal, and postrenal mechanisms, which frequently coexist in the transplant setting. In HCT recipients, the occurrence of AKI impacts clinical management by limiting the use of potentially nephrotoxic agents, including calcineurin inhibitors [13, 14], with possible repercussions on the effectiveness of prophylaxis and treatment of acute graft‐versus‐host disease (aGVHD) [7, 9].

Importantly, beyond its impact on clinical management, early AKI has been consistently associated with adverse outcomes in allogeneic HCT recipients. Previous studies have demonstrated that AKI occurring in the early post‐transplant period or around engraftment is linked to increased non‐relapse mortality and reduced overall survival, supporting its role as a clinically meaningful complication rather than an isolated laboratory abnormality [15, 16]. The early post‐transplant phase represents a particularly vulnerable window, characterized by hematopoietic aplasia, hospitalization, high risk of infections, and intensive exposure to nephrotoxic agents, including conditioning regimens and calcineurin inhibitors [13, 17]. In addition, AKI in this setting may reflect systemic processes such as endothelial dysfunction and inflammation, which are also implicated in the pathogenesis of transplant‐related complications, including aGVHD [10, 18]. Therefore, early AKI should be interpreted as a marker of global clinical severity and transplant‐related toxicity, with potential implications for subsequent outcomes and therapeutic decision‐making.

Estimates of AKI incidence among HCT recipients vary substantially across studies [19, 20, 21, 22], partly due to differences in diagnostic criteria and the characteristics of the cohorts evaluated. Given the critical nature of the early post‐transplant period, this study evaluated the incidence and severity of AKI during the first 3 weeks after HCT, as well as clinical and transplant‐related factors associated with these outcomes, with implications for preventive strategies and early management.

## 2 Methods

### 2.1 Study Population

This study included 634 consecutive recipients of allogeneic HCT performed between January 2002 and December 2023 at the Bone Marrow Transplant Center of the Brazilian National Cancer Institute (INCA), Rio de Janeiro, Brazil. Pediatric, adolescent, and adult patients who received GvHD prophylaxis with calcineurin inhibitors (cyclosporine or tacrolimus) were eligible, and only the first eligible transplant was considered for recipients undergoing multiple procedures. GvHD prophylaxis was based on calcineurin inhibitors (tacrolimus or cyclosporine) combined with methotrexate (MTX) or mycophenolate mofetil (MMF) plus post‐transplant cyclophosphamide (PTCy), according to institutional protocols. In selected cases, particularly in unrelated or haploidentical transplantation, antithymocyte globulin (ATG) was used as in vivo T‐cell depletion. Data were retrospectively collected from electronic and paper medical records and institutional transplant registries, including demographic information, clinical characteristics of recipients and donors, and transplant‐related variables, such as year of transplantation, age, sex, weight, race/ethnicity, underlying disease, donor type, HLA compatibility, stem cell source, conditioning regimen, use of total body irradiation, and GVHD prophylaxis strategies, as well as baseline laboratory parameters (aminotransferases, albumin, and hematocrit) and the type of calcineurin inhibitor used. The study was approved by the local Research Ethics Committee of INCA and conducted in accordance with the Declaration of Helsinki.

### 2.2 Definitions

AKI was defined according to the *Kidney Disease: Improving Global Outcomes* (KDIGO) 2012 criteria [12], based exclusively on changes in serum creatinine in HCT recipients (stage 1: ≥ 1.5 to 1.9 × baseline or ≥ 0.3 mg/dL increase; stage 2: 2.0 to 2.9 × baseline; stage 3: ≥ 3.0 × baseline or ≥ 4.0 mg/dL). Baseline creatinine was defined as the lowest stable value observed for at least five consecutive days prior to transplantation, in order to exclude transient fluctuations. AKI occurrence and severity were assessed during the early post‐transplant period, from day +1 to day +21, corresponding to the median length of initial hospitalization. When more than one AKI episode occurred within this interval, only the first episode was considered for analysis. AKI staging followed KDIGO definitions (stages 1–3), and urine output criteria were not included due to the retrospective design of the study and the lack of consistently available data over the extended study period.

aGVHD was diagnosed and graded based on clinical assessment of target organ involvement, including skin, gastrointestinal tract, and liver, as documented in the medical records by the attending transplant team. Given the extended study period, diagnoses and grading were performed according to institutional criteria aligned with internationally accepted consensus guidelines in use at the time of transplantation. In the earlier years of the cohort, aGVHD grading followed the classical consensus criteria described by Przepiorka et al. [23] based on the 1994 Consensus Conference on Acute GVHD Grading, incorporating organ‐specific severity and integration into an overall grade (I–IV), whereas following the implementation of updated consensus criteria around 2016, more recent cases were classified according to contemporary frameworks, including those proposed by the Mount Sinai Acute GVHD International Consortium (MAGIC) [24]. Information on aGVHD occurrence, date of diagnosis, and severity grade was collected for events occurring within the first 100 days after HCT, and the maximum overall grade observed during this period was considered for analysis. No retrospective reclassification was performed, and all diagnoses and gradings reflect contemporaneous clinical assessments.

Plasma concentrations of calcineurin inhibitors (cyclosporine or tacrolimus) were obtained from routine institutional clinical care records, as the study had a retrospective design and did not involve additional sampling for research purposes. Drug levels were measured according to standard clinical practice, and data collected between day +1 and day +21 after HCT were used for analysis. For the calculation of pharmacokinetic parameters, including median trough concentrations, percentage of supratherapeutic levels, median dose (mg/kg/day), and median concentration‐to‐dose (C/D) ratio, only values obtained prior to the occurrence of AKI were considered. Supratherapeutic levels were defined as cyclosporine > 300 μg/L and tacrolimus > 15 ng/mL.

Clinical variables related to recipients, transplant characteristics, and laboratory parameters were collected as summarized in Table 1. Laboratory variables, including AST, ALT, hematocrit, and albumin, were selected based on their potential relevance to the pharmacokinetics of calcineurin inhibitors. Hepatic enzymes may reflect variations in CYP3A‐mediated metabolism, albumin levels are associated with drug binding and distribution, and hematocrit is known to influence whole‐blood concentrations of calcineurin inhibitors due to its high affinity for erythrocytes. These parameters were therefore included to explore their potential association with calcineurin inhibitor exposure and clinical outcomes, particularly AKI.

**TABLE 1:** Baseline clinical, demographic, and transplant‐related characteristics associated with AKI in HCT recipients.

Variables	All Recipients (*N* = 634)	AKI Any stage vs. No AKI				AKI 2–3 stage vs. No AKI + stage 1			
		AKI Any stage (*n* = 346)	No AKI (*n* = 288)	HR (95% CI)	*p*	AKI Stage 2–3 (*n* = 93)	No AKI + stage 1 (*n* = 541)	HR (95% CI)	*p*
Year of transplantation									
2019–2023	175 (27.6)	87 (25.1)	88 (30.6)	1		14 (15.1)	161 (29.8)	1	
2014–2018	147 (23.2)	***88 (25.4)***	***59 (20.5)***	***1.36 (1.01–1.83)***	***0.043***	15 (16.1)	132 (24.4)	1.44 (0.70–2.98)	0.326
2009–2013	119 (18.8)	64 (18.5)	55 (19.1)	1.32 (0.95–1.82)	0.096	***20 (21.5)***	***99 (18.3)***	***2.49 (1.26–4.95)***	***0.009***
2002–2008	193 (30.4)	***107 (30.9)***	***86 (29.9)***	***1.41 (1.06–1.87)***	***0.020***	***44 (47.3)***	***149 (27.5)***	***3.46 (1.89–6.35)***	***< 0.001***
Adult	429 (67.7)	245 (70.8)	184 (63.9)	1		53 (57.0)	376 (69.5)	1	
Pediatric < 18	205 (32.3)	***101 (29.2)***	***104 (36.1)***	***0.75 (0.60–0.95)***	***0.017***	40 (43.0)	165 (30.5)	1.41 (0.93–2.12)	0.105
Age group									
1–11 year	109 (17.2)	***45 (13.0)***	***64 (22.2)***	***0.54 (0.38–0.76)***	***< 0.001***	18 (19.4)	91 (16.8)	0.76 (0.43–1.32)	0.328
12–17 year	82 (12.9)	48 (13.9)	34 (11.8)	0.93 (0.67–1.29)	0.646	18 (19.4)	64 (11.8)	1.20 (0.69–2.09)	0.528
18–39 year	232 (36.6)	136 (39.3)	96 (33.3)	1		40 (43.0)	192 (35.5)	1	
40–59 year	173 (27.3)	92 (26.6)	81 (28.1)	0.81 (0.62–1.06)	0.123	***15 (16.1)***	***158 (29.2)***	***0.45 (0.25–0.82)***	***0.009***
> 60 year	38 (6.0)	25 (7.2)	13 (4.5)	1.14 (0.74–1.74)	0.557	2 (2.2)	36 (6.7)	0.31 (0.08–1.29)	0.109
Weight median	62.3 (9.0–144.0)	***65.0 (9.0–136.5)***	***60.0 (11.5–144.0)***	***1.01 (1.00–1.01)***	***0.001***	59.1 (9.00–120.0)	63.0 (11.50–144.0)	1.00 (0.99–1.00)	0.336
Male	373 (58.8)	198 (57.2)	175 (60.8)	1		55 (59.1)	318 (58.8)	1	
Female	261 (41.2)	148 (42.8)	113 (39.2)	1.07 (0.86–1.32)	0.551	38 (40.9)	223 (41.2)	0.98 (0.65–1.48)	0.915
Color/race									
White	350 (55.2)	176 (50.9)	174 (60.4)	1		50 (53.8)	300 (55.5)	1	
Brown	226 (35.6)	***134 (38.7)***	***92 (31.9)***	***1.26 (1.01–1.58)***	***0.045***	35 (37.6)	191 (35.3)	1.15 (0.75–1.77)	0.524
Black	58 (9.1)	36 (10.4)	22 (7.6)	1.26 (0.88–1.80)	0.206	8 (8.6)	50 (9.2)	0.99 (0.47–2.09)	0.982
Primary disease									
Non‐malignant	82 (12.9)	47 (13.6)	35 (12.2)	1		13 (14.0)	69 (12.8)	1	
Malignant	552 (87.1)	299 (86.4)	253 (87.8)	0.89 (0.65–1.21)	0.455	80 (86.0)	472 (87.2)	0.87 (0.48–1.56)	0.636
Stem cell source									
BM	445 (70.2)	238 (68.8)	207 (71.9)	1		70 (75.3)	375 (69.3)	1	
PB	189 (29.8)	108 (31.2)	81 (28.1)	1.15 (0.92–1.45)	0.219	23 (24.7)	166 (30.7)	0.82 (0.51–1.31)	0.405
Donor type									
MRD	367 (57.9)	203 (58.7)	164 (56.9)	1		61 (65.6)	306 (56.6)	1	
MURD	146 (23.0)	82 (23.7)	64 (22.2)	0.94 (0.73–1.22)	0.653	22 (23.7)	124 (22.9)	0.85 (0.52–1.39)	0.525
MMURD	37 (5.8)	20 (5.8)	17 (5.9)	0.93 (0.59–1.47)	0.755	3 (3.2)	34 (6.3)	0.47 (0.15–1.51)	0.208
HAPLO	84 (13.2)	41 (11.8)	43 (14.9)	0.76 (0.54–1.06)	0.109	***7 (7.5)***	***77 (14.2)***	***0.45 (0.20–0.98)***	***0.044***
Conditioning									
RIC	220 (34.7)	124 (35.8)	96 (33.3)	1		30 (32.3)	190 (35.1)	1	
MAC	414 (65.3)	222 (64.2)	192 (66.7)	0.90 (0.72–1.12)	0.357	63 (67.7)	351 (64.9)	1.05 (0.68–1.62)	0.836
TBI									
No	354 (55.8)	198 (57.2)	156 (54.2)	1		51 (54.8)	303 (56.0)	1	
Yes	280 (44.2)	148 (42.8)	132 (45.8)	0.86 (0.70–1.07)	0.177	42 (45.2)	238 (44.0)	0.97 (0.65–1.46)	0.889
aGVHD									
No	239 (39.9)	130 (40.4)	109 (39.4)	1		41 (50.6)	198 (38.2)	1	
Yes	360 (60.1)	192 (59.6)	168 (60.6)	0.89 (0.71–1.11)	0.299	***40 (49.4)***	***320 (61.8)***	***0.59 (0.38–0.92)***	***0.019***
ALT > 56 U/L									
No	394 (70.0)	214 (67.9)	180 (72.6)	1		48 (57.8)	346 (72.1)	1	
Yes	169 (30.0)	101 (32.1)	68 (27.4)	1.22 (0.97–1.55)	0.095	***35 (42.2)***	***134 (27.9)***	***1.90 (1.23–2.94)***	***0.004***
AST > 40 U/L									
No	328 (58.2)	168 (53.3)	160 (64.3)	1		36 (43.4)	292 (60.7)	1	
Yes	236 (41.8)	***147 (46.7)***	***89 (35.7)***	***1.37 (1.10–1.71)***	***0.005***	47 (56.6)	***189 (39.3)***	***2.06 (1.33–3.17)***	***0.001***
Albumin > 3,5 g/dL									
No	294 (52.6)	***191 (61.0)***	***103 (41.9)***	***1***		***57 (68.7)***	***237 (49.8)***	***1***	
Yes	265 (47.4)	***122 (39.0)***	***143 (58.1)***	***0.53 (0.42–0.67)***	***< 0.001***	***26 (31.3)***	***239 (50.2)***	***0.39 (0.25–0.62)***	***< 0.001***
Hematocrit (%) > 25									
No	282 (50.0)	165 (52.4)	117 (47.0)	1		41 (49.4)	241 (50.1)	1	
Yes	282 (50.0)	150 (47.6)	132 (53.0)	0.87 (0.70–1.08)	0.207	42 (50.6)	240 (49.9)	0.97 (0.63–1.49)	0.890
Calcineurin Inhibitor									
CSA	563 (88.8)	306 (88.4)	257 (89.2)	1		85 (91.4)	478 (88.4)	1	
TAC	71 (11.2)	40 (11.6)	31 (10.8)	0.99 (0.71–1.38)	0.955	8 (8.6)	63 (11.6)	0.72 (0.35–1.50)	0.384

*Note:* Data are presented as *n* (%) or median (minimum–maximum). Associations are reported as hazard ratios (HR) with 95% confidence intervals (95% CI). AKI was classified as any stage (1–3) or severe (stages 2–3), considering only the first AKI episode. aGVHD was defined as any grade (I–IV) and was considered *not applicable* in patients without neutrophil engraftment. Albumin and hematocrit were dichotomized by the cohort median; missing laboratory data were ≤ 12% and reflect the retrospective design. Bold and italic values indicate statistically significant results.

Abbreviations: aGVHD, acute graft‐versus‐host disease; AKI, acute kidney injury; ALT, alanine aminotransferase; AST, aspartate aminotransferase; BM, bone marrow; CI, confidence interval; CNI, calcineurin inhibitor; CSA, cyclosporine; HAPLO, haploidentical donor; HCT, hematopoietic cell transplantation; HR, hazard ratio; MAC, myeloablative conditioning; MMURD, mismatched unrelated donor; MRD, matched related donor; MURD, matched unrelated donor; PB, peripheral blood; RIC, reduced‐intensity conditioning; TAC, tacrolimus; TBI, total body irradiation.

Conditioning regimens reflected a long study period and were therefore heterogeneous. For analytical purposes, regimens were classified according to intensity as myeloablative conditioning (MAC) or reduced‐intensity conditioning (RIC). The most frequently used MAC regimens included busulfan–cyclophosphamide (Bu4Cy) and cyclophosphamide combined with total body irradiation (Cy–TBI), with or without antithymocyte globulin (ATG), whereas the most common RIC regimens consisted of fludarabine‐based combinations (e.g., FluCy, FluMel) and reduced‐dose busulfan‐based regimens (e.g., Bu2Flu, Bu2Cy), with or without ATG or TBI.

### 2.3 Statistical Analysis

Continuous variables were summarized as median (range) and categorical variables as *n* (%), with group comparisons performed using Mann–Whitney, chi‐squared, or Fisher's exact tests, as appropriate. The cumulative incidence of AKI was estimated based on the first event within 21 days after HCT (day +1 to day +21). All descriptive, univariable, and multivariable analyses were conducted in parallel for AKI of any stage and moderate‐to‐severe AKI (stages 2–3). For analyses focusing on AKI stages 2–3, the reference group was defined as patients with no AKI and those with AKI stage 1. Year of transplantation was categorized into four groups (2002–2008, 2009–2013, 2014–2018, and 2019–2023) to account for temporal changes in transplant practices and supportive care over time.

Associations between clinical, demographic, transplant‐related, laboratory, and pharmacokinetic variables and AKI risk were assessed using Cox proportional hazards models, with time to first AKI as the outcome. Univariable models were initially fitted for both outcomes; variables associated at *p* < 0.20 were considered for multivariable adjustment, together with prespecified clinically relevant covariates (age group, year of transplantation, conditioning intensity, total body irradiation, and calcineurin inhibitor type), which were retained regardless of statistical significance. Separate multivariable models were fitted for any‐stage AKI and AKI stages 2–3, using a broader specification for the former and a more parsimonious one for the latter. Collinearity, confounding, estimate stability, and proportional hazards assumptions were assessed using standard diagnostics, including Schoenfeld residuals.

Pharmacokinetic summary measures (median plasma levels, percentage of supratherapeutic days, median dose, and concentration‐to‐dose ratio) were calculated using pre‐AKI data only. In contrast, LOESS smoothing was used to explore temporal patterns of calcineurin inhibitor plasma levels using all available measurements between day +1 and day +21, stratified by AKI status, without restricting values in the AKI group to the pre‐event period. Missing laboratory data were ≤ 12% and handled by complete‐case analysis. All tests were two‐sided, with *p* < 0.05, and analyses were performed using RStudio.

## 3 Results

### 3.1 Baseline Characteristics, AKI and aGVHD Incidence, and Univariable Associations in HCT Recipients

The study included 634 HCT recipients who underwent transplantation between 2002 and 2023. Most patients were adults (67.7%) and male (58.8%), and transplants were predominantly performed for malignant diseases (87.1%). Bone marrow was the main stem cell source (70.2%), and most procedures involved matched related donors (57.9%) and myeloablative conditioning (65.3%), with total body irradiation used in 44.2% cases. Cyclosporine was the most frequently used calcineurin inhibitor (88.8%). Baseline laboratory abnormalities were common, including hypoalbuminemia in 52.6% and elevated aminotransferases in up to 41.8% of patients. Detailed clinical, demographic, and transplant‐related characteristics are presented in Table 1. Additional analyses stratified by pediatric/adult status and age groups are presented in Supplementary Table S1. These data highlight important differences across age strata, including variation in underlying diseases (*p* < 0.001), with lymphoid malignancies and acute leukemias being more frequent in younger patients, whereas myeloid neoplasms predominate in older groups. Differences were also observed in transplant‐related characteristics, particularly regarding conditioning intensity, with a higher proportion of reduced‐intensity conditioning in older patients (52.6% in those ≥ 60 years), and stem cell source.

aGVHD occurred in 60.1% of the cohort, as presented in Table 1. Among affected patients, the distribution of severity grades showed that grade II was the most frequent (53.1%), followed by grade I (18.3%), grade III (18.1%), and grade IV (10.6%). Notably, grades II–IV, which are typically considered clinically significant and often require systemic corticosteroid therapy, accounted for the majority of cases. Age‐stratified analyses (Supplementary Table S1) demonstrated variability in aGVHD occurrence across age groups, with increasing frequency observed in older patients (*p* = 0.030). These findings provide additional clinical characterization of aGVHD in the study population.

**FIGURE 1:**
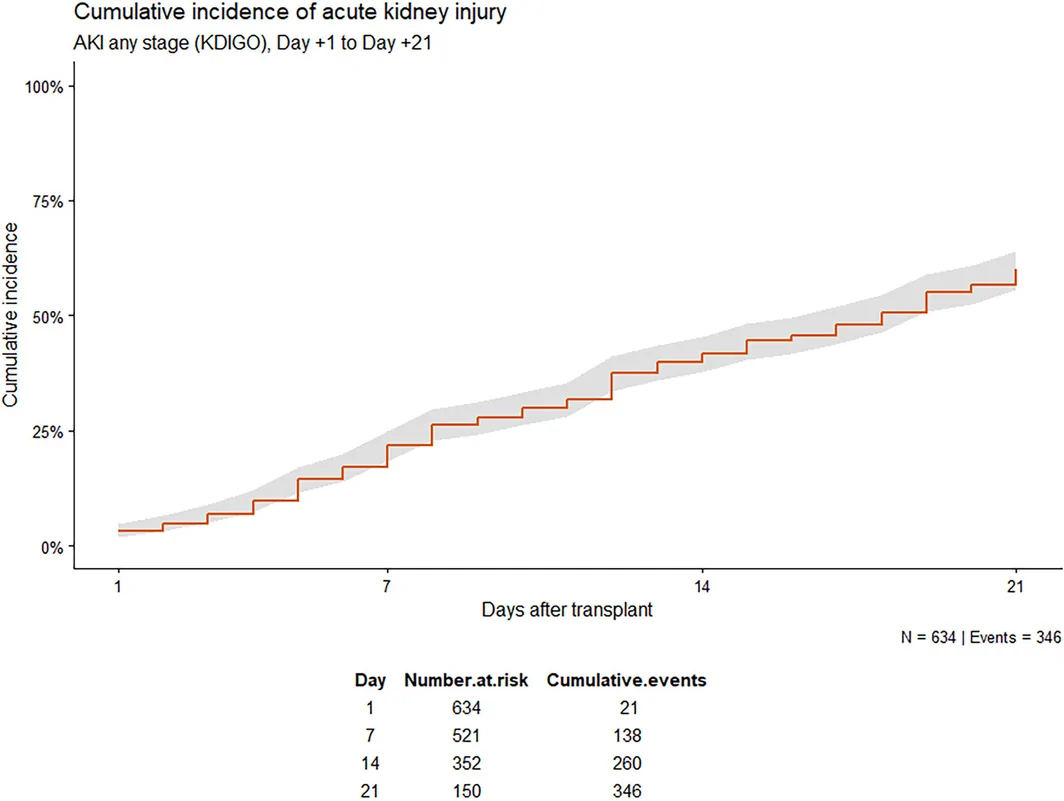
Cumulative incidence of AKI in HCT recipients. First AKI event, considering any AKI stage, among HCT recipients during the first 21 days after HCT (D + 1–D + 21).

**FIGURE 2:**
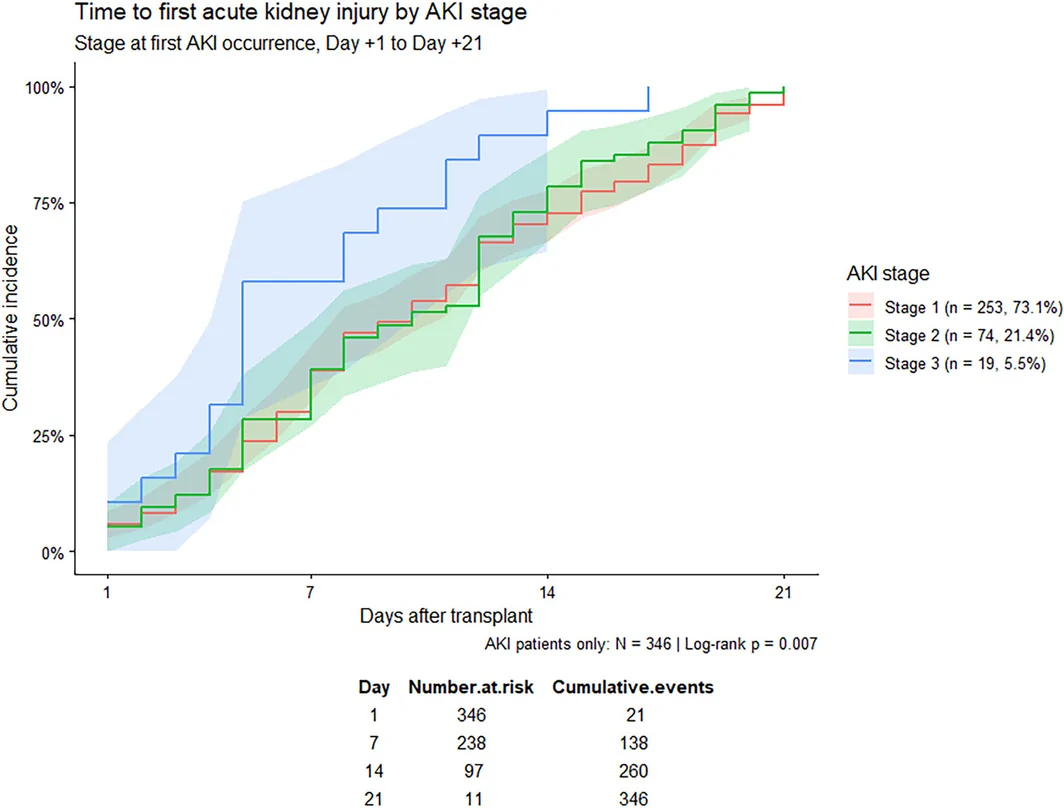
Cumulative incidence of AKI by stage in HCT recipients. First AKI event among HCT recipients during the first 21 days after HCT (from D + 1 to D + 21), stratified according to the stage.

During the first 21 days after HCT (from D + 1 to D + 21), AKI showed a high cumulative incidence, reaching approximately 55% by day 21, with 346 events among 634 recipients (Figure 1). The incidence increased progressively over time, with about 25% of patients developing AKI by day 7 and more than 40% by day 14. Among patients who developed AKI, stage 1 was the most frequent first event (73.1%), followed by stage 2 (21.4%) and stage 3 (5.5%) (Figure 2). The median time to AKI was 12 days (IQR: 8–14.8) overall, 12 days (IQR: 7–13.8) for stage 1, 10 days (IQR: 5–14) for stage 2, and 5 days (IQR: 4–10) for stage 3. Time‐to‐event analysis demonstrated significant differences in the temporal distribution according to AKI stage, with largely overlapping curves for stages 1 and 2 and an earlier occurrence of stage 3 despite its lower frequency (log‐rank *p* = 0.007).

In the univariate cumulative incidence analysis of acute kidney injury, stratified by clinical, demographic, and transplant‐related characteristics, earlier categories of year of transplantation were associated with a higher cumulative incidence of AKI, both for any‐stage AKI and for stages 2–3, with a progressively increased risk in periods prior to 2014 (Table 1). Age influenced the cumulative incidence of AKI, with a lower incidence observed among pediatric patients, particularly those aged 1–11 years, whereas patients aged 40–59 years showed a lower cumulative incidence of stage 2–3 AKI compared with the reference group. Higher body weight was associated with a greater cumulative incidence of any‐stage AKI. Among sociodemographic variables, brown race was associated with a higher cumulative incidence of any‐stage AKI. Transplant‐related characteristics, including stem cell source, donor type, conditioning intensity, use of total body irradiation, and type of calcineurin inhibitor, were not associated with differences in the cumulative incidence of AKI. Regarding clinical and laboratory variables, elevated AST and ALT levels were associated with a higher cumulative incidence of AKI, particularly for stages 2–3, whereas albumin levels > 3.5 g/dL were consistently associated with a lower cumulative incidence of AKI, both for any‐stage and for more severe AKI. The presence of acute graft‐versus‐host disease was associated with a lower cumulative incidence of stage 2–3 AKI.

### 3.2 Pharmacologic Determinants of AKI Related to Calcineurin Inhibitors

Among cyclosporine‐treated recipients, pre‐AKI pharmacokinetic exposure differed between patients who developed any‐stage acute kidney injury and those without AKI (Table 2). Patients with AKI showed higher median pre‐injury trough levels (274.8 vs. 260.9 ng/mL) and a greater proportion of supratherapeutic days (50% vs. 33%). Median daily dose (mg/kg/day) and the concentration‐to‐dose (C/D) ratio in the pre‐AKI period did not differ consistently between groups. These differences were not observed when considering stage 2–3 AKI only. Among tacrolimus‐treated recipients, pre‐AKI trough levels, proportion of supratherapeutic days, daily dose, and C/D ratio were similar between patients with and without AKI, both for any‐stage AKI and for stage 2–3 AKI. Complete comparisons of pre‐AKI pharmacokinetic parameters are presented in Table 2.

**TABLE 2:** Pre‐AKI calcineurin inhibitor pharmacokinetic exposure and risk of acute kidney injury in allogeneic HCT recipients (*N* = 634).

Variables	AKI Any stage vs. No AKI				AKI 2–3 stage vs. No AKI + stage 1			
Cyclosporine (*n* = 563)	AKI Any stage (*n* = 306)	No AKI (*n* = 257)	HR (95% CI)	*p*	AKI Stage 2–3 (*n* = 85)	No AKI + stage 1 (*n* = 478)	HR (95% CI)	*p*
Median trough level (pre‐AKI)	***274.80 (199.00–368.25)***	***260.90 (212.20–324.67)***	***1.11 (1.05–1.17)***	***< 0.001***	269.00 (201.57–398.75)	264.50 (205.65–341.00)	1.04 (0.95–1.13)	0.427
% of supratherapeutic days pre‐AKI	***0.50 (0.00–1.00)***	***0.33 (0.16–0.60)***	***1.06 (1.02–1.10)***	***0.005***	0.50 (0.00–1.00)	0.333 (0.00–0.66)	1.05 (0.98–1.13)	0.196
Median dose mg/kg/day (pre‐AKI)	***3.01 (2.86–3.20)***	***3.07 (2.81–3.62)***	***0.93 (0.87–0.99)***	***0.019***	3.03 (2.93–3.19)	3.03 (2.81–3.41)	1.02 (0.92–1.14)	0.657
Median C/D ratio pre‐AKI	92.63 (62.92–127.45)	84.28 (58.86–122.53)	1.00 (1.00–1.00)	0.655	82.34 (61.41–127.29)	87.84 (60.90–124.55)	1.00 (1.00–1.00)	0.781

Tacrolimus (*n* = 71)	AKI Any stage (*n* = 40)	No AKI (*n* = 31)	HR (95% CI)	*p*	AKI Stage 2–3 (*n* = 8)	No AKI + stage 1 (*n* = 63)	HR (95% CI)	*p*
Median trough level (pre‐AKI)	6.14 (4.73–8.49)	8 (6–10.9)	0.95 (0.88–1.03)	0.217	7.13 (5.43–8.15)	7.34 (4.71–9.81)	1.04 (0.88–1.23)	0.661
% of supratherapeutic days pre‐AKI	6.25 (0–23.6)	0 (0–28.6)	0.95 (0.82–1.10)	0.496	22.5 (8.33–29.2)	0 (0–22.2)	1.21 (0.97–1.52)	0.091
Median dose mg/kg/day (pre‐AKI)	0.020 (0.018–0.030)	0.020 (0.017–0.027)	1.00 (0.99–1.01)	0.845	0.022 (0.016–0.032)	0.020 (0.017–0.027)	0.98 (0.88–1.11)	0.790
Median C/D ratio pre‐AKI	319 (159–516)	380 (163–592)	0.93 (0.83–1.04)	0.217	372 (166–633)	319 (160–544)	1.12 (0.89–1.40)	0.344

*Note:* Supratherapeutic days indicate the percentage of days with trough levels above the therapeutic range. Bold and italic values indicate statistically significant results.

Abbreviations: AKI, acute kidney injury; C/D ratio, concentration‐to‐dose ratio; CI, confidence interval; CSA, cyclosporine; HCT, hematopoietic cell transplantation; HR, hazard ratio; Pre‐AKI, period before the first AKI episode; TAC, tacrolimus.

**FIGURE 3:**
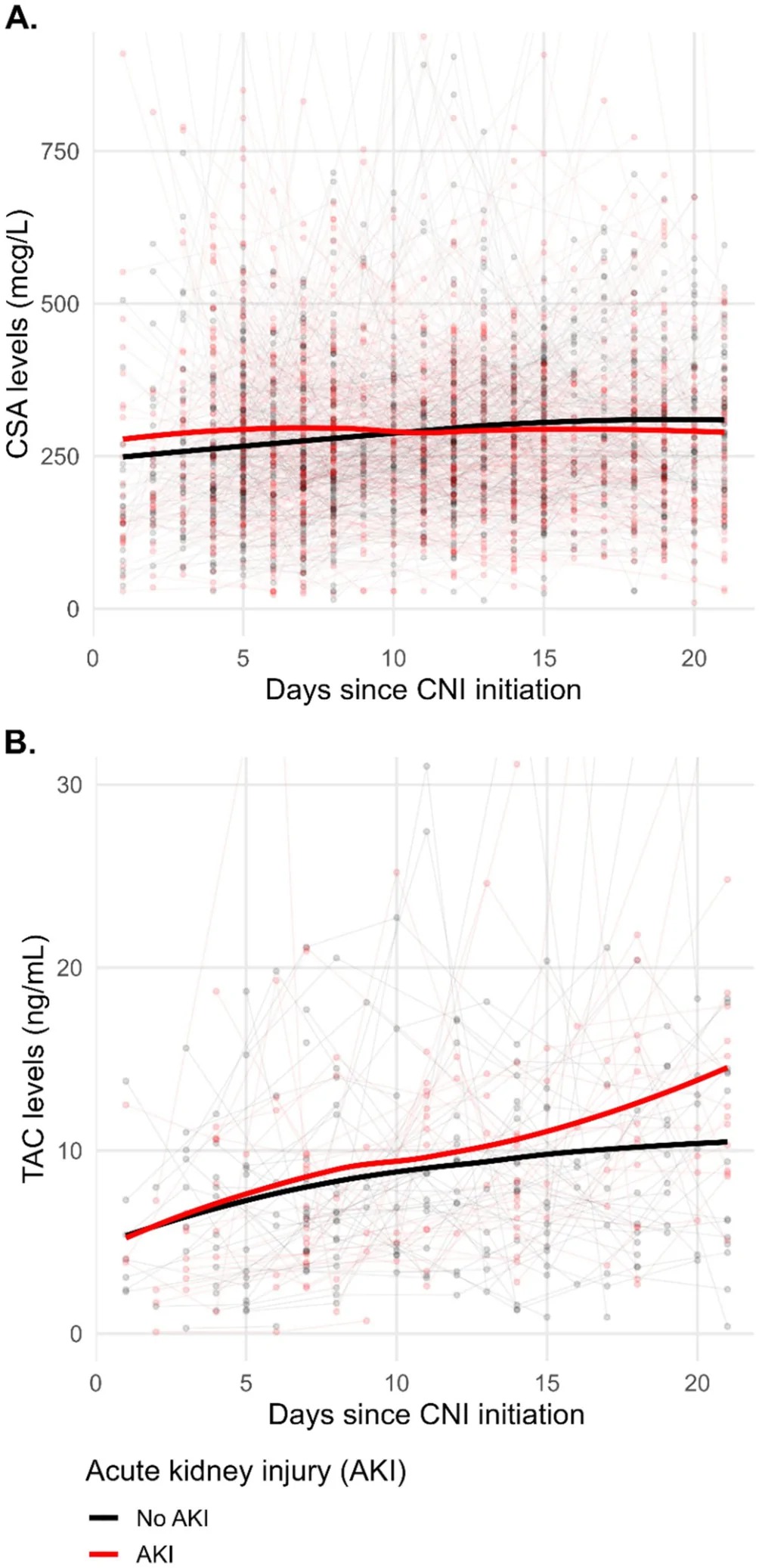
LOESS‐smoothed curves of CSA (A) and TAC (B) plasma levels according to acute kidney injury (AKI; stages 1–3) during the first 21 days after allogeneic HCT.

Longitudinal analysis of plasma levels during the first 21 days revealed distinct patterns between cyclosporine and tacrolimus when comparing patients with and without AKI (Figure 3). For cyclosporine, plasma levels were generally higher among patients who developed AKI, particularly after day 10 following calcineurin inhibitor initiation; however, an inversion of the curves was observed thereafter, with lower levels in the AKI group compared with patients without AKI (Figure 3A). For tacrolimus, the curves showed substantial overlap during the early treatment period, followed by a progressive separation from approximately day 7 onward, resulting in markedly higher plasma levels among patients with AKI by day 21 (Figure 3B). Overall, these findings indicate an earlier occurrence of AKI in cyclosporine‐treated recipients compared with tacrolimus, along with distinct longitudinal plasma level trajectories between the two calcineurin inhibitors.

### 3.3 Multivariable Analysis of Factors Associated With AKI

In multivariable Cox proportional hazards models evaluating time to first AKI within the first 21 days after HCT, several variables remained independently associated with the risk of AKI (Table 3). For any‐stage AKI, higher body weight was associated with an increased risk (HR per +1 Kg 1.01, 95% CI 1.00–1.02), and elevated AST levels (> 40 U/L) were independently associated with a higher risk of AKI (HR 1.58, 95% CI 1.17–2.12). In contrast, albumin levels > 3.5 g/dL were consistently associated with a lower risk of AKI (HR 0.56, 95% CI 0.44–0.71). In addition, recipients classified as brown race had a higher risk of any‐stage AKI compared with white recipients (HR 1.34, 95% CI 1.05–1.71). No independent associations were observed for conditioning intensity, total body irradiation, donor type, or calcineurin inhibitor type in this model.

**TABLE 3:** Multivariable analysis of factors associated with AKI in HCT recipients.

Variable	Any‐AKI HR (95% CI)	*p*	Stages 2–3 AKI HR (95% CI)	*p*
Age group (ref.: 18–39 year)				
1–11 year	0.80 (0.49–1.30)	0.373	0.93 (0.51–1.72)	0.828
12–17 year	1.11 (0.74–1.65)	0.620	1.26 (0.68–2.33)	0.461
40–59 year	0.84 (0.63–1.13)	0.258	***0.50 (0.27–0.96)***	***0.036***
> 60 year	1.46 (0.91–2.32)	0.113	0.48 (0.11–2.05)	0.322
Year of transplantation (ref.: 2019–2023)				
2014–2018	***1.40 (1.00–1.94)***	***0.047***	1.39 (0.66–2.94)	0.383
2009–2013	1.41 (0.96–2.06)	0.077	***2.89 (1.33–6.27)***	***0.007***
2002–2008	1.38 (0.95–2.02)	0.091	***3.68 (1.75–7.72)***	***< 0.001***
Weight (per + 1 kg)	***1.01 (1.00–1.02)***	***0.018***	—	—
Conditioning (MAC vs. RIC)	0.95 (0.72–1.25)	0.711	0.70 (0.41–1.18)	0.181
TBI (Yes vs. No)	0.97 (0.72–1.29)	0.815	0.94 (0.57–1.57)	0.826
Calcineurin inhibitor (CSA vs. TAC)	0.98 (0.68–1.42)	0.917	0.83 (0.36–1.93)	0.670
ALT > 56 U/L	0.99 (0.73–1.34)	0.924	1.33 (0.76–2.33)	0.311
AST > 40 U/L	***1.58 (1.17–2.12)***	***0.003***	***1.93 (1.08–3.44)***	***0.025***
Albumin > 3.5 g/dL	***0.56 (0.44–0.71)***	***< 0.001***	***0.42 (0.26–0.70)***	***< 0.001***
Donor type (ref.: MRD)				
MURD	0.99 (0.59–1.65)	0.961	—	—
MMURD	0.98 (0.73–1.31)	0.870	—	—
HAPLO	0.84 (0.54–1.29)	0.421	—	—
Sel‐reported race/color (ref.: White)				
Brown	***1.34 (1.05–1.71)***	***0.020***	—	—
Black	1.30 (0.89–1.91)	0.179	—	—

*Note:* Values are hazard ratios (HR) with 95% confidence intervals (95% CI) from multivariable Cox models. Separate models were fitted for any acute kidney injury (AKI; KDIGO stages 1–3) and severe AKI (KDIGO stages 2–3), considering only the first AKI episode within 21 days after HCT. Bold and italic values indicate statistically significant results.

Abbreviations: AKI, acute kidney injury; ALT, alanine aminotransferase; AST, aspartate aminotransferase; CI, confidence interval; CSA, cyclosporine; HAPLO, haploidentical donor; HCT, hematopoietic cell transplantation; HR, hazard ratio; KDIGO, Kidney Disease: Improving Global Outcomes; MAC, myeloablative conditioning; MMURD, mismatched unrelated donor; MRD, matched related donor; MURD, matched unrelated donor; RIC, reduced‐intensity conditioning; TAC, tacrolimus; TBI, total body irradiation.

In the multivariable model restricted to severe AKI (KDIGO stages 2–3), earlier years of transplantation remained strongly associated with increased risk, particularly for transplants conducted between 2009 and 2013 (HR 2.89, 95% CI 1.33–6.27) and 2002–2008 (HR 3.68, 95% CI 1.75–7.72), compared with the most recent period. Elevated AST levels were also independently associated with severe AKI (HR 1.93, 95% CI 1.08–3.44), whereas albumin levels > 3.5 g/dL were associated with a substantially lower risk (HR 0.42, 95% CI 0.26–0.70). Patients aged 40–59 years had a lower risk of AKI stages 2–3 compared with the reference age group. Other prespecified covariates, including conditioning intensity, total body irradiation, and calcineurin inhibitor type, were not independently associated with severe AKI.

## 4 Discussion

In this cohort of recipients, the incidence of AKI within the first 21 days after HCT was approximately 55%, confirming the high frequency of this complication in the early post‐transplant period. Among patients who developed AKI, approximately 27% presented with moderate to severe forms (KDIGO stages 2–3), underscoring the clinical relevance of AKI severity in this setting. Both the occurrence and severity of AKI were associated with multiple factors. Baseline clinical and laboratory characteristics, including age, body weight, hypoalbuminemia, and elevated aminotransferases, were associated with the risk of AKI of any stage as well as with more severe forms. Among transplant‐related variables, the year of transplantation showed a consistent association, particularly with stages 2–3 AKI. In addition, pharmacologic aspects related to calcineurin inhibitor exposure, including plasma levels and the proportion of days with supratherapeutic concentrations, were associated with AKI development, placing early post‐HCT kidney injury within a framework of interacting clinical, transplant‐related, and pharmacologic factors that warrant integrated analysis.

Previous studies have reported highly variable incidences of post‐HCT AKI according to the duration of observation. Most cohorts evaluated renal outcomes over extended periods, commonly up to 100 days, as in the studies by Mima et al. (15.7%) [25], Gan et al. (29.2%) [26], Menezes et al. (32%) [27], Matsuoka et al. (34.6%) [28], Sun et al. (54.9%) [29], Abramson et al. and Madsen et al. (64%) [13, 30] and Andronesi et al. (68.9%) [31], whereas Sakaguchi et al. reported an incidence of 64.9% over 200 days [32]. In contrast, fewer investigations have focused on shorter time frames, with Bhasin et al. (23%) [33] and Vergara‐Cadavid et al. (33%) [15] evaluating AKI within the first 30 days after transplantation. Within this context, our cumulative incidence of approximately 55% by day 21 highlights the substantial burden of renal injury occurring during the very early post‐transplant phase. By specifically targeting the initial hospitalization period, our study captures a critical window characterized by intense conditioning‐related toxicity, early calcineurin inhibitor exposure, hemodynamic instability, infectious complications, and frequent need for intensive supportive care. This narrow temporal focus may partly explain the high incidence observed despite the shorter follow‐up and supports the clinical relevance of early AKI as a marker of vulnerability in this phase. Taken together, the marked variability across studies reflects differences in observation windows, AKI definitions, surveillance strategies, patient case mix, and institutional practices, underscoring that reported incidences are highly context‐dependent and reinforcing the importance of standardized methodologies when interpreting and comparing post‐HCT renal outcomes.

In this study, the main factors associated with an increased risk of AKI in the early post‐HCT period showed strong convergence with previously reported findings. Older age was identified as a risk factor in multiple cohorts [13, 27, 33], in agreement with our results, although this effect was not uniformly observed for more severe outcomes across studies. From an anthropometric perspective, the association between higher body weight and increased AKI risk observed in our analysis was consistent with the concept of metabolic vulnerability, although other cohorts did not demonstrate independent associations with body mass index or body surface area after adjustment [13, 25, 29]. From a pharmacological standpoint, we observed higher pre‐injury cyclosporine exposure and a greater proportion of supratherapeutic days among patients who developed AKI, converging with studies that linked supratherapeutic calcineurin inhibitor levels to AKI occurrence [15, 31]. In addition, the higher incidence of AKI among recipients classified as brown race highlights the heterogeneity observed in analyses evaluating race and ethnicity, in which effect estimates have varied substantially across populations [13, 15]. Notably, earlier years of transplantation in our cohort were independently associated with a higher risk of AKI in our cohort, an association that has not been consistently reported in previous studies and may reflect progressive improvements in supportive care, therapeutic monitoring, and overall peri‐transplant management over time.

Age was identified as a factor associated with AKI in our cohort, with heterogeneous effects across different age groups. A lower risk of AKI was observed in pediatric patients, which may be related, at least in part, to age‐related pharmacokinetic differences, as children tend to exhibit higher metabolic clearance of calcineurin inhibitors [34, 35], potentially resulting in lower systemic exposure and reduced nephrotoxicity. In contrast, patients aged 40–59 years showed a lower risk of severe AKI (stages 2–3) compared with the reference group (18–39 years) in the adjusted analysis. This finding may be related to differences in clinical and transplant‐related characteristics across age groups. In age‐stratified analyses, patients aged 40–59 years presented lower exposure to total body irradiation, a higher proportion of matched related donors, and differences in self‐reported race/color distribution, with a lower frequency of categories associated with increased AKI risk in our model. In contrast, conditioning intensity was similar between these groups, suggesting that this factor alone does not account for the observed association. These findings indicate that the relationship between age and AKI risk in the early post‐HCT period is likely multifactorial, reflecting differences in case‐mix, transplant characteristics, and baseline clinical profile. Therefore, this result should be interpreted with caution.

Regarding factors associated with a reduced risk of AKI, previous studies have demonstrated a lower incidence of AKI in specific GVHD prophylaxis settings, particularly in T‐cell–depleted transplants or in regimens based on tacrolimus alone, compared with combinations containing sirolimus or post‐HCT cyclophosphamide [13, 30]. These findings suggest that differences in immunosuppressive strategies and calcineurin inhibitor exposure may play a central role in shaping renal outcomes in the early post‐transplant period. Although atypical forms of GVHD with renal involvement have been described in the literature [36] and may be associated with an increased risk of AKI, this mechanism does not appear to explain the findings in our cohort. In contrast, the inverse association between aGVHD and severe AKI observed in the univariable analysis in our study is more consistent with the pharmacological balance of calcineurin inhibitor exposure, in which lower CNI levels may increase the risk of GVHD while reducing nephrotoxicity, whereas higher levels may provide more effective GVHD prophylaxis at the expense of increased renal injury, supporting a trade‐off between immunosuppressive efficacy and toxicity. However, this association was not sustained in the multivariable analysis, suggesting that it may reflect a non‐independent relationship. Furthermore, the relationship between GVHD, CNI exposure, and AKI is likely dynamic, as the onset of GVHD may lead to subsequent intensification of immunosuppressive therapy, while severe GVHD may contribute to volume depletion and prerenal kidney injury. Therefore, these findings should be interpreted within a complex temporal framework, in which pharmacological modulation and clinical complications may interact over time.

Conversely, HCT‐related variables were not independently associated with AKI in our cohort, as also reported in other studies, including conditioning intensity and exposure to TBI [25, 26, 29], donor type [13, 25, 30], graft source [15, 28, 30], and calcineurin inhibitor type [26, 33]. Taken together, these results reinforce that, in the early post‐HCT period, the occurrence of AKI is more strongly determined by the balance between immunosuppressive efficacy and calcineurin inhibitor–related nephrotoxicity, combined with individual clinical vulnerability, than by structural characteristics of the transplant.

Lower cyclosporine levels observed after day 10 in patients who developed AKI likely reflect therapeutic adjustments following renal dysfunction, including dose reduction or temporary discontinuation of calcineurin inhibitors in clinical practice. This pattern suggests a reverse causality effect, in which AKI leads to subsequent changes in drug exposure rather than lower exposure being protective. In contrast, tacrolimus exhibited a different longitudinal pattern, with overlapping levels between AKI and non‐AKI groups during the early post‐transplant period, followed by a progressive separation over time, resulting in higher levels among patients with AKI at later time points. These findings suggest distinct temporal relationships between calcineurin inhibitor exposure and AKI, with cyclosporine‐associated AKI occurring earlier and tacrolimus‐related differences in exposure becoming more apparent over time. Therefore, longitudinal comparisons of calcineurin inhibitor levels should be interpreted with caution, particularly in the context of toxicity‐driven dose modifications.

In our cohort, baseline albumin levels > 3.5 g/dL were among the most consistent predictors of lower AKI risk during the very early post‐HCT period, both for any‐stage AKI (HR 0.56) and for AKI stages 2–3 (HR 0.42), whereas AST levels > 40 U/L were independently associated with an increased risk of AKI (any AKI: HR 1.58; AKI stages 2–3: HR 1.93). These findings are consistent with evidence from other cohorts, in which higher albumin levels were associated with a lower incidence of AKI, either as a continuous effect (Abramson [13]: HR 0.59 per 1 g/dL increase) or based on specific cutoff values (Sun [29]: albumin > 40.6 g/L associated with lower risk of AKI stages 2–3, HR 0.515; Vergara‐Cadavid [15]: albumin < 3.4 g/dL associated with increased risk, HR 2.04; Madsen [30]: protective association in univariable analysis, *p* = 0.015). From a pathophysiological perspective, hypoalbuminemia likely reflects a higher inflammatory burden and reduced clinical reserve, in addition to contributing to decreased effective intravascular volume and greater vulnerability to renal hypoperfusion [37, 38]. Conversely, elevated AST levels may indicate hepatocellular dysfunction, directly affecting calcineurin inhibitor biotransformation and favoring increased systemic exposure and subsequent renal injury [39].

This study has some limitations that should be acknowledged. Its retrospective design relies on the quality and completeness of medical records, which may have restricted the systematic inclusion of some clinically relevant variables. Early post‐transplant complications, such as cytomegalovirus reactivation, infections, thrombotic microangiopathy, sinusoidal obstruction syndrome, as well as variables including sepsis, use of nephrotoxic antimicrobial agents, hypertension, and volume depletion related to mucositis or gastrointestinal GVHD, are recognized contributors to AKI but were not specifically addressed in the present analysis. This reflects the inherent complexity of evaluating a large retrospective cohort of HCT recipients over a period exceeding two decades, in which multiple clinical, therapeutic, and institutional factors evolve over time. The evaluation of long‐term outcomes, such as overall survival and non‐relapse mortality, or the impact of early AKI on these endpoints, would be more challenging in this cohort due to the heterogeneity of allogeneic HCT recipients, encompassing diverse underlying diseases and transplant characteristics. Such analyses were beyond the scope of the present study and would require more homogeneous populations and dedicated study designs and should be addressed in future investigations. In addition, the historical predominance of cyclosporine use compared with tacrolimus reflects institutional practice changes over time and may have limited direct comparisons between calcineurin inhibitors. Furthermore, the absence of more comprehensive anthropometric measures, such as body mass index or body surface area, limited the assessment of body composition, as body weight was the only consistently available variable across the cohort. Despite these limitations, the large cohort size, institutional representativeness, and robust analytical approach strengthen the clinical relevance and reliability of the present findings.

## 5 Conclusions

This study demonstrates a high early incidence of AKI in allogeneic HCT recipients (55% by D + 21), with more than one‐quarter of cases reaching moderate‐to‐severe stages and earlier onset among severe events. Elevated AST, higher body weight, brown race, and transplantation in earlier years of the study period were independently associated with increased AKI risk, whereas baseline albumin > 3.5 g/dL was consistently protective. Higher pre‐AKI cyclosporine exposure and a greater proportion of supratherapeutic days were associated with AKI, while transplant‐related characteristics showed no independent effect. Collectively, these findings clarify the incidence, severity, and main clinical and pharmacological determinants of early AKI and support targeted monitoring and individualized immunosuppression during the initial post‐transplant period.

## Author Contributions

**Luiz Carlos da Costa‐Junior:** conceptualization, data curation, formal analysis, investigation, methodology project administration, resources, software, supervision, validation, visualization writing – original draft, writing – review and editing. **Rita de Cássia Barbosa da Silva Tavares:** conceptualization, data curation, investigation, methodology, supervision writing – original draft, writing – review and editing. **Marina Izu:** data curation, investigation, methodology, writing – review and editing; **Simone Cunha Maradei:** data curation, investigation, writing – review and editing. **Maria Claudia Rodrigues Moreira:** investigation, resources, supervision, writing – review and editing. **Marta Colares Nogueira:** formal analysis, data interpretation, supervision, writing – review and editing. **Décio Lerner:** conceptualization, funding acquisition, project administration, resources supervision, writing – review and editing. **Fabrício Guimarães Bino:** investigation, methodology, writing – original draft, writing – review and editing. **Patrícia Moriel:** conceptualization, funding acquisition, project administration, resources supervision, writing – review and editing. **Teresa de Souza Fernandez:** conceptualization, funding acquisition, project administration, resources supervision, writing – review and editing. **Paulo Caleb Júnior Lima Santos:** conceptualization, formal analysis, funding acquisition, methodology project administration, resources, supervision, validation, visualization, writing – original draft, writing – review and editing.

## Funding

This work was supported by Coordenação de Aperfeiçoamento de Pessoal de Nível Superior (001), Fundação de Amparo à Pesquisa do Estado de São Paulo (2024/07284‐9) and Fundação Carlos Chagas Filho de Amparo à Pesquisa do Estado do Rio de Janeiro (26/201.218/2022).

## Conflicts of Interest

Conselho Nacional de Desenvolvimento Científico e Tecnológico (CNPQ); 001 – Coordenação de Aperfeiçoamento de Pessoal de Nível Superior (CAPES); Fundação de. Amparo à Pesquisa do Estado de São Paulo (FAPESP 2024/07284‐9). Fundação Carlos Chagas Filho de Amparo à Pesquisa do Estado do Rio de Janeiro—FAPERJ, T.d.S.F. (FAPERJ/E‐26/201.218/2022). The authors declare there are no conflicts of interest related to this study.

## Supporting information


**Table S1:** Baseline characteristics of hematopoietic cell transplantation recipients stratified by age group and pediatric/adult status.

## Data Availability

The data that support the findings of this study are available on request from the corresponding author. The data are not publicly available due to privacy or ethical restrictions.
