# Supplementary material for: Early Acute Kidney Injury in Allogeneic Hematopoietic Cell Transplantation: Incidence, Severity, and Associated Factors in a 634‐Recipient Cohort
Source: Eur J Haematol. 2026 May 21;117(4):981–94. doi: 10.1111/ejh.70227 (PMC13542945; doi:10.1111/ejh.70227)
Supplement: Supplementary file 1 [file EJH-117-981-s001.docx]

| Supplementary Table 1. Baseline characteristics of hematopoietic cell transplantation recipients stratified by age group and pediatric/adult status | | | | | | | | | | |
| --- | --- | --- | --- | --- | --- | --- | --- | --- | --- | --- |
| **Variables** | **Total** | **Pediatric <18** | **Adult** | ***P*** | **Age group** | | | | | ***P*** |
|  |  |  |  |  | **1–11** | **12–17** | **18–39** | **40–59** | **≥60** |  |
|  |  |  |  |  |  |  |  |  |  |  |
| **Demographics** |  |  |  |  |  |  |  |  |  |  |
|  |  |  |  |  |  |  |  |  |  |  |
| Sex |  |  |  |  |  |  |  |  |  |  |
| Male | 373 (58.8%) | 135 (65.9%) | 238 (55.5%) | 0.017 | 67 (61.5%) | 58 (70.7%) | 142 (61.2%) | 93 (53.8%) | 13 (34.2%) | 0.002 |
| Female | 261 (41.2%) | 70 (34.1%) | 191 (44.5%) |  | 42 (38.5%) | 24 (29.3%) | 90 (38.8%) | 80 (46.2%) | 25 (65.8%) |  |
| Weight, kg | 62.4 (48.0–75.0) | 35.4 (24.0–50.2) | 69.5 (60.0–80.0) | <0.001 | 24.9 (18.7–33.0) | 48.0 (40.0–57.0) | 67.0 (59.0–77.0) | 73.0 (62.7–82.0) | 65.0 (60.0–72.0) | <0.001 |
| Color/race |  |  |  |  |  |  |  |  |  |  |
| White | 350 (55.2%) | 107 (52.2%) | 243 (56.6%) | 0.024 | 54 (49.5%) | 43 (52.4%) | 118 (50.9%) | 109 (63.0%) | 26 (68.4%) | 0.008 |
| Brown | 226 (35.6%) | 86 (42.0%) | 140 (32.6%) |  | 46 (42.2%) | 37 (45.1%) | 90 (38.8%) | 45 (26.0%) | 8 (21.1%) |  |
| Black | 58 (9.1%) | 12 (5.9%) | 46 (10.7%) |  | 9 (8.3%) | 2 (2.4%) | 24 (10.3%) | 19 (11.0%) | 4 (10.5%) |  |
|  |  |  |  |  |  |  |  |  |  |  |
|  |  |  |  |  |  |  |  |  |  |  |
| **Transplant-related** |  |  |  |  |  |  |  |  |  |  |
|  |  |  |  |  |  |  |  |  |  |  |
| Year of transplantation |  |  |  |  |  |  |  |  |  |  |
| 2002–2008 | 193 (30.4%) | 61 (29.8%) | 132 (30.8%) | 0.592 | 35 (32.1%) | 21 (25.6%) | 81 (34.9%) | 54 (31.2%) | 2 (5.3%) | 0.002 |
| 2009–2013 | 119 (18.8%) | 38 (18.5%) | 81 (18.9%) |  | 17 (15.6%) | 15 (18.3%) | 48 (20.7%) | 35 (20.2%) | 4 (10.5%) |  |
| 2014–2018 | 147 (23.2%) | 54 (26.3%) | 93 (21.7%) |  | 33 (30.3%) | 19 (23.2%) | 45 (19.4%) | 39 (22.5%) | 11 (28.9%) |  |
| 2019–2023 | 175 (27.6%) | 52 (25.4%) | 123 (28.7%) |  | 24 (22.0%) | 27 (32.9%) | 58 (25.0%) | 45 (26.0%) | 21 (55.3%) |  |
| Stem cell source |  |  |  |  |  |  |  |  |  |  |
| BM | 445 (70.2%) | 183 (89.3%) | 262 (61.1%) | <0.001 | 103 (94.5%) | 68 (82.9%) | 157 (67.7%) | 96 (55.5%) | 21 (55.3%) | <0.001 |
| PB | 189 (29.8%) | 22 (10.7%) | 167 (38.9%) |  | 6 (5.5%) | 14 (17.1%) | 75 (32.3%) | 77 (44.5%) | 17 (44.7%) |  |
| HLA |  |  |  |  |  |  |  |  |  |  |
| MRD | 367 (57.9%) | 87 (42.4%) | 280 (65.3%) | <0.001 | 51 (46.8%) | 29 (35.4%) | 137 (59.1%) | 129 (74.6%) | 21 (55.3%) | <0.001 |
| MUD | 146 (23.0%) | 68 (33.2%) | 78 (18.2%) |  | 33 (30.3%) | 30 (36.6%) | 51 (22.0%) | 23 (13.3%) | 9 (23.7%) |  |
| MMUD | 37 (5.8%) | 18 (8.8%) | 19 (4.4%) |  | 11 (10.1%) | 5 (6.1%) | 13 (5.6%) | 8 (4.6%) |  |  |
| Haploidentical | 84 (13.2%) | 32 (15.6%) | 52 (12.1%) |  | 14 (12.8%) | 18 (22.0%) | 31 (13.4%) | 13 (7.5%) | 8 (21.1%) |  |
| Conditioning |  |  |  | 0.038 |  |  |  |  |  |  |
| RIC | 220 (34.7%) | 59 (28.8%) | 161 (37.5%) |  | 31 (28.4%) | 24 (29.3%) | 86 (37.1%) | 59 (34.1%) | 20 (52.6%) | 0.062 |
| MAC | 414 (65.3%) | 146 (71.2%) | 268 (62.5%) |  | 78 (71.6%) | 58 (70.7%) | 146 (62.9%) | 114 (65.9%) | 18 (47.4%) |  |
| TBI |  |  |  |  |  |  |  |  |  |  |
| No | 354 (55.8%) | 72 (35.1%) | 282 (65.7%) | <0.001 | 42 (38.5%) | 22 (26.8%) | 130 (56.0%) | 129 (74.6%) | 31 (81.6%) | <0.001 |
| Yes | 280 (44.2%) | 133 (64.9%) | 147 (34.3%) |  | 67 (61.5%) | 60 (73.2%) | 102 (44.0%) | 44 (25.4%) | 7 (18.4%) |  |
|  |  |  |  |  |  |  |  |  |  |  |
|  |  |  |  |  |  |  |  |  |  |  |
| **Clinical** |  |  |  |  |  |  |  |  |  |  |
|  |  |  |  |  |  |  |  |  |  |  |
| Primary disease |  |  |  |  |  |  |  |  |  |  |
| Malignant | 552 (87.1%) | 170 (82.9%) | 382 (89.0%) | 0.043 | 90 (82.6%) | 67 (81.7%) | 192 (82.8%) | 167 (96.5%) | 36 (94.7%) | <0.001 |
| Non-malignant | 82 (12.9%) | 35 (17.1%) | 47 (11.0%) |  | 19 (17.4%) | 15 (18.3%) | 40 (17.2%) | 6 (3.5%) | 2 (5.3%) |  |
| Disease classification |  |  |  |  |  |  |  |  |  |  |
| AML | 146 (23.0%) | 36 (17.6%) | 110 (25.6%) | <0.001 | 17 (15.6%) | 13 (15.9%) | 57 (24.6%) | 45 (26.0%) | 14 (36.8%) | <0.001 |
| ALL | 179 (28.2%) | 106 (51.7%) | 73 (17.0%) |  | 58 (53.2%) | 42 (51.2%) | 47 (20.3%) | 28 (16.2%) | 4 (10.5%) |  |
| CML | 80 (12.6%) | 7 (3.4%) | 73 (17.0%) |  | 2 (1.8%) | 5 (6.1%) | 31 (13.4%) | 40 (23.1%) | 2 (5.3%) |  |
| MDS/MPN | 75 (11.8%) | 12 (5.9%) | 63 (14.7%) |  | 10 (9.2%) | 2 (2.4%) | 21 (9.1%) | 30 (17.3%) | 12 (31.6%) |  |
| Lymphoid malig. | 66 (10.4%) | 9 (4.4%) | 57 (13.3%) |  | 3 (2.8%) | 5 (6.1%) | 35 (15.1%) | 19 (11.0%) | 4 (10.5%) |  |
| MM | 6 (0.9%) |  | 6 (1.4%) |  |  |  | 1 (0.4%) | 5 (2.9%) |  |  |
| Bone marrow failure | 70 (11.0%) | 32 (15.6%) | 38 (8.9%) |  | 16 (14.7%) | 15 (18.3%) | 32 (13.8%) | 5 (2.9%) | 2 (5.3%) |  |
| Other non-malignant | 12 (1.9%) | 3 (1.5%) | 9 (2.1%) |  | 3 (2.8%) |  | 8 (3.4%) | 1 (0.6%) |  |  |
|  |  |  |  |  |  |  |  |  |  |  |
|  |  |  |  |  |  |  |  |  |  |  |
| **Early complications** |  |  |  |  |  |  |  |  |  |  |
|  |  |  |  |  |  |  |  |  |  |  |
| aGVHD |  |  |  |  |  |  |  |  |  |  |
| No | 239 (39.9%) | 86 (44.8%) | 153 (37.6%) | 0.112 | 52 (51.0%) | 28 (36.4%) | 93 (42.5%) | 56 (33.7%) | 10 (28.6%) | 0.030 |
| Yes | 360 (60.1%) | 106 (55.2%) | 254 (62.4%) |  | 50 (49.0%) | 49 (63.6%) | 126 (57.5%) | 110 (66.3%) | 25 (71.4%) |  |
| AKI (any) |  |  |  |  |  |  |  |  |  |  |
| No | 288 (45.4%) | 104 (50.7%) | 184 (42.9%) | 0.077 | 64 (58.7%) | 34 (41.5%) | 96 (41.4%) | 81 (46.8%) | 13 (34.2%) | 0.018 |
| Yes | 346 (54.6%) | 101 (49.3%) | 245 (57.1%) |  | 45 (41.3%) | 48 (58.5%) | 136 (58.6%) | 92 (53.2%) | 25 (65.8%) |  |
| AKI (stage 2–3) |  |  |  |  |  |  |  |  |  |  |
| No | 541 (85.3%) | 165 (80.5%) | 376 (87.6%) | 0.024 | 91 (83.5%) | 64 (78.0%) | 192 (82.8%) | 158 (91.3%) | 36 (94.7%) | 0.013 |
| Yes | 93 (14.7%) | 40 (19.5%) | 53 (12.4%) |  | 18 (16.5%) | 18 (22.0%) | 40 (17.2%) | 15 (8.7%) | 2 (5.3%) |  |
|  |  |  |  |  |  |  |  |  |  |  |
|  |  |  |  |  |  |  |  |  |  |  |
| **Prophylaxis** |  |  |  |  |  |  |  |  |  |  |
|  |  |  |  |  |  |  |  |  |  |  |
| CNI |  |  |  |  |  |  |  |  |  |  |
| Cyclosporine | 563 (88.8%) | 180 (87.8%) | 383 (89.3%) | 0.678 | 99 (90.8%) | 68 (82.9%) | 203 (87.5%) | 163 (94.2%) | 30 (78.9%) | 0.014 |
| Tacrolimus | 71 (11.2%) | 25 (12.2%) | 46 (10.7%) |  | 10 (9.2%) | 14 (17.1%) | 29 (12.5%) | 10 (5.8%) | 8 (21.1%) |  |
|  |  |  |  |  |  |  |  |  |  |  |
| **Abbreviations:** HCT, hematopoietic cell transplantation; AKI, acute kidney injury; aGVHD, acute graft-versus-host disease; CNI, calcineurin inhibitor; BM, bone marrow; PB, peripheral blood; HLA, human leukocyte antigen; MRD, matched related donor; MUD, matched unrelated donor; MMUD, mismatched unrelated donor; RIC, reduced-intensity conditioning; MAC, myeloablative conditioning; TBI, total body irradiation; MDS, myelodysplastic syndrome; MPN, myeloproliferative neoplasm.  **Data are presented as n (%) or median (interquartile range).** Comparisons were performed using the chi-square or Fisher’s exact test for categorical variables and nonparametric tests for continuous variables.  **Primary diseases were standardized from free-text entries and grouped into predefined diagnostic categories.** Lymphoid malignancies included Hodgkin lymphoma, non-Hodgkin lymphoma, and adult T-cell leukemia/lymphoma (ATLL). Myelodysplastic/myeloproliferative neoplasms (MDS/MPN) comprised myelodysplastic syndromes (MDS), chronic myelomonocytic leukemia (CMML), juvenile myelomonocytic leukemia (JMML), and myelofibrosis. Non-malignant diseases were categorized into bone marrow failure syndromes, including aplastic anemia, Fanconi anemia, pure red cell aplasia, and Diamond–Blackfan anemia, and other non-malignant disorders, including paroxysmal nocturnal hemoglobinuria, congenital neutropenia (Kostmann syndrome), Wiskott–Aldrich syndrome, and adrenoleukodystrophy. The classification was based on clinically meaningful groupings consistent with transplant registries and hematologic literature, with minor adaptations according to the distribution of diagnoses in the study population. | | | | | | | | | | |
